# Significance of boost radiotherapy in early invasive ductal breast cancer with ductal carcinoma *in situ* component under negative surgical margins

**DOI:** 10.1093/jrr/rrab103

**Published:** 2021-10-27

**Authors:** Naoko Shimizu, Miyako Myojin, Motoshi Tamura, Noriaki Nishiyama, Katsushige Yamashiro, Yuichi Yuyama, Yutaka Okazaki, Yasuhiro Suzuki, Masato Takahashi

**Keywords:** primary breast cancer, ductal carcinoma in situ (DCIS) component, negative margin, ipsilateral breast tumor recurrence (IBTR), boost irradiation

## Abstract

We hypothesize that there is a risk of ipsilateral breast tumor recurrence (IBTR) in surgical margin-free invasive ductal carcinoma (IDC) in the presence of ductal carcinoma *in situ* (DCIS) component affecting surgical margins in early stage. From 1990 to 2014, 343 patients with IDC in which the DCIS component constitute have received radiotherapy (RT) following breast-conserving surgery (BCS). All patients received whole breast irradiation with a prescribed dose of 50 Gy in 20 fractions (four times a week). This one-arm cohort with boost RT (253 patients) was compared for IBTR with a non-cohort group receiving no boost RT because of freedom from positive margins (90 patients). Median observation months were 98 (boost group) vs 119 (no boost group), respectively. The 15-year local recurrence-free survival (LRFS) rates were 98.5% and 85.6% in the boost and no boost groups, respectively (Cox proportional hazards model univariate analysis; p = 0.013, HR 0.13). Similarly, for other background factors, there was a significant difference in the LRFS between age groups. The 15-year LRFS rate was 91.8% in patients aged 45 years or younger and 94.6% in patients older than 46 years (p = 0.031, HR 0.21), respectively. Only these two factors were independently significant in Cox proportional hazards model multivariate analysis. IBTR risk in margin-free IDC with DCIS component was independently decreased by boost RT in the cohort setting. Tumor size, extensive intraductal component (EIC), boost dose, the presence of lymph node (LN) metastasis and hormonal therapy were not IBTR risk factors in this study.

## INTRODUCTION

One of the treatments following whole breast radiotherapy (WBRT) after lumpectomy in early-stage breast cancer is the delivery of radiotherapy (RT) boost treatment, whereby an additional four- to- eight fractions (F) are added to the surgical bed to eliminate residual tumor cells. Multiple clinical trials [1–3] identified this practice as effecting a modest but statistically significant reduction in ipsilateral breast tumor recurrence (IBTR) for invasive breast cancer in all age groups. To date, no similar phase 3 trials have been conducted for ductal carcinoma *in situ* (DCIS). Pooled, retrospective data from 10 Western academic institutions indicated the benefit of boost treatment in the context of negative margins in 2016 [4]. In the same year, re-analysis for prognostic factors in a European Organization for Research and Treatment of Cancer (EORTC) ‘boost no boost’ trial demonstrated an association between DCIS and an increased risk of IBTR at 20-year follow-up. Furthermore, additional DCIS served as an indication for long-term follow-up [5]. In Japan, no long-term follow-up results have been derived in this regard.

**Fig. 1:**
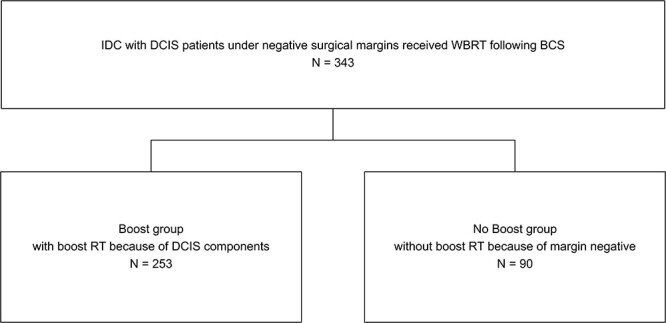
Summarizations of the study design for pT1-T2 IDC patients with DCIS component under negative surgical margins. BCS = breast-conserving surgery; RT = radiotherapy, WBRT = whole breast radiation therapy.

The rate of invasive ductal carcinoma (IDC) coexisting pathologically with DCIS is high, with roughly two- thirds of IDC being accompanied by DCIS [6]. Since many DCIS cases are not palpable and the boundaries ambiguous, it is often difficult to accurately assess the spread of DCIS. Investigating each pathological report and specimen prior to postoperative RT, we formulated the hypothesis that there is a risk of local recurrence for early IDC with DCIS components, even if the margin is negative on pathological examination after breast-conserving surgery (BCS). As part of this cohort study, boost irradiation treatment was applied following WBRT in patients with IDC accompanied by DCIS.

In the past decade, RT technology for WBRT has changed from simulator planning to computed tomography (CT) planning. After introducing CT treatment plans for WBRT without any changes to the electron boost method, we followed prescription doses and RT schedules to achieve the equivalent dose indicated the simulator plan. In this way, it was possible to evaluate the long-term results over a period of 20 years using multi-institutional collaborations.

Although our cohort was a small-sized population, we prospectively accumulated patients treated with boost irradiation therapy for marginal-free IDC on DCIS component and report the results of long-term observations herein.

## MATERIALS AND METHODS

### Study design

In the present study, a one-arm prospective cohort with boost RT was compared for IBTR with a controlled IDC group without boost RT (Fig. 1). The control group comprised historical and same-period patients after 1990 treated without boost RT because of the presence of negative margins, according to the guide at that time. At our institution, electron boost had been performed only in the patients with positive margins since 1975 with the policy of not uniformly boosting for patients with negative margins. On the other hand, patients in the cohort group received boost RT because they had IDC with DCIS component. The results of this study were reviewed retrospectively. Long-term observations were made for both the boost and non-boost groups to examine the presence of IBTR, to compare LRFS and review background factors.

All procedure were in accordance with the ethical standards of the responsible committee on human experimentation and with the Helsinki Declaration of 1964 and later versions. This research was approved by the institutional review board at the National Hospital Organization Hokkaido Cancer Center and Keiyukai Sapporo Hospital, Japan**.**

### Patients

We analyzed patients who underwent RT after BCS from May 1990 to February 2014, although entry of the boost group began in March 1998. Patients had been diagnosed as having IDC with negative margins and with a geometric factor affecting margins such as IDC with DCIS component. It was clarified in the literatures that negative margin width associated with local recurrence was <2 mm [7, 8]. Since patients with IDC accompanied by DCIS were examined in the present study, a margin of ≥2 mm was defined as a margin negative. All patients were treated with partial resection. Following surgery, patients who received chemotherapy prior to starting RT were excluded. We also excluded patients with bilateral simultaneous breast cancer and patients with a history of cancer treatment elsewhere.

### RT planning method and RT technique

Tangential RT for WBRT planned by fluoroscopic simulation was prescribed at 2.5 Gy per fraction at the isocenter, placed at equidistance in the anterior–posterior direction of two portal beams. The fractionated schedule was a total of 50 Gy in 20 fractions for 5 weeks. To realize similar mean doses delivered to the clinical target volume (delineation for the mammary glands and nipple and surrounding soft tissue) for patients via a CT simulator after 2009, the prescribed dose was 47.5 Gy in 20 fractions for 5 weeks at the centroid of the planning target volume. The dose was realized for 95% of patients to achieve 47.5–52.5 Gy in 20 fractions at the two-dimensional center of two portal beams. To decrease the maximum dose to less than 2.6 Gy per fraction, a field-in-field plan [9], which involved a weight optimization technique for three-to-five beams in the radiation treatment planning system (Pinnacle3), was adopted. WBRT was performed using 4MV X-ray. The appropriate energy of electron beam was selected from 4 MeV, 6 MeV, 9 MeV, and 12 MeV. WBRT and boost RT were performed four times a week. For the dose fractionation of ipsilateral WBRT, 50 Gy/20 fractions/5 weeks (four times a week, instructed dose at the center of tangential irradiation by the simulator) have been used since about half a century at anonymous University Hospital, our university of origin.

All patients underwent ultrasonography to detect the depth and location of the lumpectomy cavity. The boost RT field was determined based on sonographic imaging and surgical scar by adding 2- to 3-cm margins in all directions. The electron beam energy of boost RT treatment was determined by referencing depth to the lumpectomy cavity and the distance from the body surface to the lung.

Boost treatment was performed at a dose of 10 Gy in 4 fractions for 1 week or 15 Gy in 6 fractions for 1.5 weeks, optionally. The two boost doses were dependent on the period of treatment. Although a boost dose of 10 Gy/4 fractions/5-7 days was used initially in this study, a boost dose of 15 Gy/6 fractions/9 days (13.55 Gy_10_ at α/β = 10) was used after 2009, which is comparable to the 16 Gy/8 fractions/10 days (13.43 Gy_10_ at α/β = 10) used in the EORTC report [10].

### Statistical analysis

A chi-squared test was performed to compare the characteristics of patients, i.e. age (≤45 years and ≥ 46 years), pathological T stage, hormonal therapy, the presence of lymph node (LN) metastasis, the presence of an extensive intraductal component (EIC), chemotherapy, hormone receptor status, HER2 status and margin distance. The Mann–Whitney U test was used to compare median age and follow-up time between the no-boost group and the boost group.

IBTR was defined as ipsilateral tumor growth near the tumor bed or in the residual mammary gland. If the tumor was an IDC, it was considered a recurrent tumor, even if the pathological features changed to another type of IDC. LRFS was defined as the survival time from the start of RT to ipsilateral intramammary recurrence. The cumulative LRFS from the date of starting RT to the date of IBTR was calculated using the Kaplan–Meier method. The statistical differences of LRFS rates were derived using the log-rank test and the Cox proportional hazard model for univariate analysis. Multivariate analyses for LRFS rates were performed using the stepwise forward and backward methods in the Cox regression hazard model. When p < 0.05, a statistically significant difference in all statistical tests was observed. All statistical analyses were performed using the IBM SPSS Statistics v. 25. software package.

## RESULTS

We examined 343 early breast cancer patients with negative margins, who had IDC with DCIS component. The non-boost group included 90 patients and the boost group included 253 patients. Among cases with boost RT, 99 patients received a 10 Gy boost, and 154 patients received a 15 Gy boost. Patient characteristics in this study are provided in Table 1. The only statistically significant difference in background factors between the boost and no boost groups was the margin distance. In the no boost group, there were many cases of ≥5 mm or more in the non-boost group significantly. The reason for the statistically significant difference was not because it was intentionally screened, but because the opinions of negative margins has changed by surgeons over time.

**Table 1:** Patients’ characteristics according to grouping by boost irradiation

Characteristic	No. (%) of Patients			P Value
	All	No Boost Group	Boost Group	
	N = 343	N = 90	N = 253	
Follow up, m	13-225	13-215	21-225	p = 0.061^*^
Median	100	119	98	
Age, y	19-80	19-78	30-80	p = 0.392^*^
Median	53	51	53	
Age, y				
≤45	88 (25.7)	28(31.1)	60 (23.7)	p = 0.17^*^^*^
≥46	255 (74.3)	62 (68.9)	193 (76.3)	
Tumor size				
pT1	280 (81.6)	84 (93.3)	196 (77.5)	p = 0.001^*^^*^
pT2	63 (18.4)	6 (6.7)	57 (22.5)	
HM therapy^*^^*^^*^				
Yes	249 (72.6)	68 (75.6)	181 (71.5)	p = 0.463^*^^*^
No	94 (27.4)	22 (24.4)	72 (28.5)	
LN metastasis				
Positive	18 (5.2)	3 (3.3)	15 (5.9)	p = 0.343^*^^*^
Negative	325 (94.8)	87 (96.7)	238 (94.1)	
EIC				
Positive	31 (9.0)	8 (8.9)	23 (9.1)	p = 0.954^*^^*^
Negative	312 (91.0)	82 (91.1)	230 (90.9)	
Nuclear grade				
Low	258 (75.2)	68 (75.6)	190 (75.1)	p = 0.996^*^^*^
Intermediate	31 (9.0)	8 (8.9)	23 (9.1)	
High	54 (15.7)	14 (15.6)	40 (15.8)	
Chemotherapy				
Yes	24 (7.0)	3 (3.3)	21 (8.3)	p = 0.149^*^^*^
No	319 (93.0)	87 (96.7)	232 (91.7)	
ER status				
Positive	261 (76.1)	62 (68.9)	199 (78.7)	p = 0.157^*^^*^
Negative	61 (17.8)	20 (22.2)	41 (16.2)	
Unknown	21 (6.1)	8 (8.9)	13 (5.1)	
PgR status				
Positive	223 (65.0)	56 (62.2)	167 (66.0)	p = 0.301^*^^*^
Negative	85 (24.8)	21 (23.3)	64 (25.3)	
Unknown	35 (10.2)	13 (14.5)	22 (8.7)	
HER2				
Positive	15 (4.3)	5 (5.5)	10 (4.0)	p = 0.894^*^^*^
Equivocal	29 (8.5)	8 (8.9)	21 (8.3)	
Negative	228 (66.5)	60 (66.7)	168 (66.4)	
Unknown	71 (20.7)	17 (18.9)	54 (21.3)	
Margin distance				
> = 2 mm - <5 mm	120 (35.0)	4 (4.4)	116 (45.8)	p < 0.001^*^^*^
> = 5 mm	223 (65.0)	86 (95.6)	137 (54.2)	

HM = hormonal; LN = lymph node; EIC = extensive intraductal components; ER = estrogen receptor; PgR = progesterone receptor.

^^*^^The Mann–Whitney U test.

^^*^^*^^A chi-squared test.

^^*^^*^^*^^Hormonal therapy for less than two years was considered as no hormonal therapy.

In the nuclear grading, nuclear grade 3 was about 15% in all groups, although it was not possible to recollect all blocks and perform central pathological analysis. However, on the intraductal component of each case, no cases were found in which the pleomorphic pattern and mitoses frequency were significantly inconsistent with the IDC’s nuclear grade determination [11]. We investigated whether nuclear grade affects local recurrence, but of the eight cases of local recurrence in this study, only one case of nuclear grade 3 was included in six cases of recurrence in the no boost group.

**Fig. 2:**
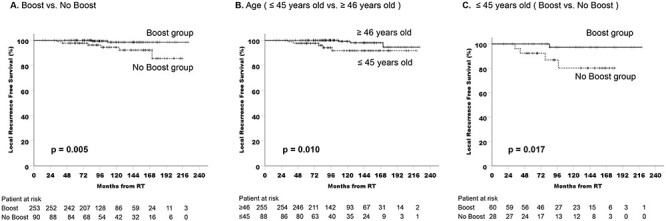
Cumulative LRFS curves for patient groups. **A.** Patient groups with boost RT or without boost RT. The 10- and 15-year LRFS rates were 98.5% and 98.5% in the boost group, and 94.4% and 85.6% in the no-boost group, respectively, there was a significant difference between them. **B.** Patient groups by age factors (≤ 45 years and ≥ 46 years). The 10- and 15-year LRFS rates were 91.8% and 91.8% in patients younger than 45 years and 99.1% and 94.6% in patients older than 46 years, respectively. There was a significant difference in the LRFS between age factors. **C.** Cumulative LRFS curves for a population of 45 years and younger patient with boost RT or without boost RT. The 10-LRFS rates were 97.2% in the boost group, and 80.2% in the no-boost group, respectively. There was a significant difference between them (p = 0.017).

The LRFS rates based on patient distribution for each clinical factor are shown in Table 2. IBTR was observed in eight cases (2.4%), 6 (6.7%) of which were in the no boost group and 2 (0.8%) of which were in the boost group. The 10- and 15-year LRFS rates were 98.5% and 98.5% in the boost group and 94.4% and 85.6% in the no boost group, respectively, indicating a significant difference between the two (log rank, p = 0.005) (Fig. 2-A). Additional background factors are shown in Table 2. There was a significant difference in LRFS based on age (≤ 45 years vs ≥ 46 years), and the 10 and 15-year LRFS rates were 91.8% and 91.8% in patients 45 years or younger and 99.1% and 94.6% in patients older than 46 years, respectively (log rank, p = 0.010) (Fig. 2-B).

**Table 2:** The results of univariate analysis for Kaplan–Meier survival curve method

(No. of Patients)	10-year LRFS (SE)	15-year LRFS (SE)	p Value	p Value
			(log-rank)	(Cox proportional univariate analysis)
Boost RT				
No (90)	94.4 (0.028)	85.6 (0.071)	p = 0.005	p = 0.015
Yes (253)	98.5 (0.011)	98.5 (0.011)		
10 Gy (99)	100 (0)	100 (0)	p = 0.366	p = 0.591
15 Gy (154)	97.9 (0.015)	97.9 (0.015)		
Age				
<=45 (88)	91.8 (0.037)	91.8 (0.037)	p = 0.010	p = 0.022
> = 46 (255)	99.1 (0.009)	94.6 (0.037)		
Tumor size				
pT1 (280)	98.1 (0.010)	94.0 (0.034)	p = 0.698	p = 0.699
pT2 (63)	94.8 (0.037)	94.8 (0.037)		
HM therapy				
Yes (249)	98.1 (0.010)	96.9 (0.015)	p = 0.571	p = 0.574
No (94)	95.6 (0.031)	88.2 (0.076)		
LN metastasis				
Positive (18)	100 (0)	100 (0)	p = 0.582	p = 0.716
Negative (325)	97.3 (0.011)	93.8 (0.030)		
EIC				
Positive (31)	89.7 (0.074)	89.7 (0.074)	p = 0.064	p = 0.088
Negative (312)	98.1 (0.010)	94.5 (0.030)		
Magin distance				
> = 2 mm- < 5 mm (120)	95.7 (0.043)	95.7 (0.043)	p = 0.405	p = 0.419
> = 5 mm (223)	97.1 (0.013)	93.4 (0.032)		

LRFS = local recurrence free survival; SE = standard error; RT = radiotherapy; HM = hormonal; LN = lymph node; EIC = extensive intraductal components.

There was no significant difference in LRFS rates between IBTR in pT1 and pT2 patients (six patients, pT1; two patients, pT2; log rank, p = 0.698) (Table 2). In addition to pathological T-stages, the presence of hormonal therapy, LN metastasis, EIC and margin distance were compared for 10- and 15-year LRFS between each of the two groups in Table 2; no significant differences between two groups were observed for any of these factors in all 343 patients. Although the no boost group included many cases with large margin distances apparently, IBTR was more common in the no boost group. As previously reported, it has been found that a difference in margin distance from 2 mm to 5 mm does not significantly affect local recurrence [12, 13], and this study also showed that margin distance did not affect local recurrence.

Statistically significant factors in multivariate analysis are shown in Table 3. Three significant factors for IBTR were derived from Cox Proportional Hazards Regression model analyses (same results were obtained by both stepwise forward and back ward methods), i.e. boost vs no boost (p = 0.012), young age (≤ 45 years vs ≥ 46 years, p = 0.031) and EIC (p = 0.049). If p-values for regression coefficients are corrected by Bonferroni adjustments in tests, p = 0.017 is the statistical significance level. Therefore, only boost RT was a significant factor in the IDC with DCIS patients under negative surgical margins.

**Table 3:** Cox proportional hazards regression multivariable analysis of IBTR (A result of forward stepwise selection method)

Characteristic	HR (95% CI)	P Value
Boost Irradiation		
No	1 [reference]	
Yes	0.13 (0.03-0.64)	0.013
Age, y		
≤45	1 [reference]	
≥46	0.21 (0.05-0.86)	0.031
EIC		
Positive	1 [reference]	
Negative	0.19 (0.04-0.99)	0.049

IBTR = ipsilateral breast tumor recurrence; HR = hazard ratio; CI = confidence interval; EIC = extensive intraductal components.

Case information for IBTR is shown in Table 4. Five of the local recurrent cases were younger than 45 years, and three of these patients died because of breast cancer with distant metastasis. Only two recurrent patients in the boost group were treated with 15 Gy in 6 fractions boost therapy. Detailed information for these two cases is summarized in Table 5. The recurrence locations were found at the margins of boost RT fields or outside the RT field. The case with recurrence outside the field indicated no histopathological relation to the initial tumor.

**Table 4:** Cases of ipsilateral breast tumor recurrence

Age (years)	Boost Dose (Gy)	pT Stage	EIC	HM Therapy	LRFS (months)	Distant metastasis
25	0	T1	−	TAM	33.9	+
31	0	T1	+	−	97.6	+
34	0	T1	+	TAM	41.5	+
57	0	T1	−	TAM	123.8	−
48	0	T1	−	−	171.4	−
43	0	T1	−	TAM	78.2	+
40	15	T2	−	TAM	84.4	−
67	15	T2	−	−	106.9	−

EIC = extensive intraductal components; HM = hormonal; TAM = Tamoxifen; LRFS = local recurrence free survival.

We also conducted an analysis only for the younger patient groups (≤ 45 years) with boost RT or without boost RT (Fig. 2-C). The 10- LRFS rates were 97.2% and 80.2% in the boost group and the no-boost group, respectively. There was a significant difference between them (p = 0.017). In the population of younger patients, the local recurrence in the no boost group tended to be observed relatively early in follow-up time. The average LRFS in younger group was 67.1 months, which was shorter than that (92.2 months) of all local recurrent patients (Table 4).

## DISCUSSION

This study was initially planned 21 years ago to verify the significance of boost RT treatment in surgical margin-negative IDC with geometric factors on IBTR risk. The factors in question included DCIS based on a review of early breast cancer surgical specimens. Although this study analyzed a small population according to limited pathological features, the research design adopted a one-arm cohort setting for patients with DCIS component, such an approach has not previously been reported. And our conclusions were drawn from long-term follow-up, as10-year survival was generally obtained within a 15- to 20-year follow-up duration.

We subsequently concluded that boost treatment could decrease (12.9%) a 15-year IBTR risk for patients with DCIS component. This indicated that IBTR risk from ‘cancer progression structure’ may be significant regardless of tumor size (pT1 or pT2). IBTR after WBRT for patients with DCIS component was statistically different in two independent factors i.e. boost vs no boost and age ≤ 45 years vs ≥ 46 years, both in single-variate and multivariate analyses.

Vrieling *et al.* showed that in the EORTC ‘boost no boost’ trial, young age and the presence of DCIS increased the risk of IBTR [5]. They derived results from subgroup analyses in a large database. This study considered long-term follow-up of pathological prognostic factors associated with a local control. Previous analyses of the EORTC study showed that young age and high-grade IDCs were linked to a high risk of local recurrence after BCS [14], and high-grade tumors showed frequent IBTR earlier during follow-up than at 5-years. Nonetheless, the relative effect of the presence of DCIS did not decrease over time. Thus, it was concluded that patients with IDC must be closely monitored, particularly during the first 5 years, whereas patients with IDC accompanied by DCIS need long-term follow-up for at least 20 years.

Accordingly, we experienced that, regardless of margin distance, the risk of IBTR depends on biological characteristics such as cancer progression structure. This cannot be explained simply as a relationship between geometric factors. In a recent study of DCIS, it was considered to be a precursor of IDC and four models were proposed to describe the progression of DCIS into invasive breast cancer [15]. However, the structure of cancer progression remains unclear in this context. In a report on DCIS growth by Faverly *et al.,* 90% of poorly differentiated lesions grew, uninterruptedly; 70% of well-differentiated lesions had a multifocal, skip pattern; 82% of skip lesions measured between 0 and 5 mm and 8% indicated skip lesions of >10 mm [16]. These DCIS progression patterns differed based on the pathological grades of DCIS; therefore, the relationship between IBTR and the pathological grade of DCIS requires further investigation. In recent years, reports on the relationship between DCIS pathological grades and local recurrence were published [17, 18]. However, it is unclear whether this DCIS classification has an impact on boost RT indication. In recent results involving ‘boost no boost’ trial, the background histological grades of IDC were not a risk factor for IBTR [5].

It is known that IBTR in IDC may follow distant metastases, and that suppression of local recurrence affects long-term prognosis [19, 20]. In our study, distant metastases occurred in four out of eight local recurrence cases. In particularly, three patients younger than 45 years died from distant metastases, none of whom received boost RT. Therefore, additional boost RT may be recommended for young patients, regardless of pathological features, as already indicated in a large-scale randomized control study [1, 5].

**Table 5:** IBTR cases in boost group

Age, y	TNM	Primary Location	RT Dose	Recurrence Location
40	T2N0M0	Right central	50Gy + boost 15Gy/6F	Right upper inner quadrant
				Boost irradiation field edge
67	T2N0M0	Right upper outer quadrant	50Gy + boost 15Gy/6F	Right central
				Out of Boost irradiation field

IBTR = ipsilateral breast tumor recurrence; RT = radiation therapy; F = fraction.

Remarkably, two of the patients in our study with IBTR who received boost RT experienced local relapse at the margin of the electron boost field (see Table 5). According to the results of a SEER survey conducted by Azu *et al.* [21] involving 318 surgeons who had treated breast cancer, physicians were unable to gain a particular indication of the histological state that was to constitute an appropriate negative margin. A randomized trial of quadrantectomy vs lumpectomy by Veronesi *et al.* demonstrated a lower rate of local recurrence in the former group [22]. Although lumpectomy was performed at a gross margin of 1 cm, 16% of those in the lumpectomy group had a positive margin. In other words, local recurrence in the lumpectomy contributed to margin status via factors such as tumor on ink and aggressive biology. Considering this fact, even if a 2-mm margin was secured, a treatment exhibiting an anti-tumor effect that surpassed tumor biology was required.

In this study’s boost group, recurrence was only 0.8%, both occurring from the boost RT margin edge or outside the margin instead of demanding stronger intensive treatment. It may be necessary to investigate a method of setting boost field.

One of the limitations of this study was a fact that too few events, i.e. eight IBTR, affected the results. Since the two cohorts were compared without matching in this study, it was difficult to obtain credibility comparable to a randomized controlled trial. The other limitation was the lack of central pathological review because this study was continued for 24 years.

Pathological sections for examination can be sliced thinner than before. We use a technique called boost to compensate for the uncertainty about the margin of histopathological judgment, which increases in accuracy with the times. As the accuracy of pathologically margin-negative techniques increases, the role of boost RT may diminish. However, if DCIS remains even a little, RT is definitely effective. This article only clarified the implications of irradiating when a margin of 2 mm is secured, that is, for a very small possibility of tumor cell retention.

In conclusion, this study indicated that the IDC patients with DCIS component should be considered the most relevant candidates for boost RT, even if the surgical margin is negative.

## CLINICAL TRIAL REGISTRATION

This research was approved by the institutional review board at the National Hospital Organization Hokkaido Cancer Center and Keiyukai Sapporo Hospital, Japan (UMIN000030052).

## PRESENTATION AT A CONFERENCE

The 80th annual meeting of the Japan Radiological Society.
